# Improving primary care in British Columbia, Canada: evaluation of a peer-to-peer continuing education program for family physicians

**DOI:** 10.1186/1472-6920-12-110

**Published:** 2012-11-09

**Authors:** Dan MacCarthy, Liza Kallstrom, Helena Kadlec, Marcus Hollander

**Affiliations:** 1 Practice Support and Quality, British Columbia Medical Association, Vancouver, BC, Canada; 2 Practice Support Program, British Columbia Medical Association, Vancouver, BC, Canada; 3 Hollander Analytical Services Ltd, Victoria, BC, Canada

**Keywords:** Primary care, Continuing education, Practice change, Evaluation, Outcomes

## Abstract

**Background:**

An innovative program, the Practice Support Program (PSP), for full-service family physicians and their medical office assistants in primary care practices was recently introduced in British Columbia, Canada. The PSP was jointly approved by both government and physician groups, and is a dynamic, interactive, educational and supportive program that offers peer-to-peer training to physicians and their office staff. Topic areas range from clinical tools/skills to office management relevant to General Practitioner (GP) practices and “doable in real GP time”. PSP learning modules consist of three half-day learning sessions interspersed with 6–8 week action periods. At the end of the third learning session, all participants were asked to complete a pen-and-paper survey that asked them to rate (a) their satisfaction with the learning module components, including the content and (b) the perceived impact the learning has had on their practices and patients.

**Methods:**

A total of 887 GPs (response rates ranging from 26.0% to 60.2% across three years) and 405 MOAs (response rates from 21.3% to 49.8%) provided responses on a pen-and-paper survey administered at the last learning session of the learning module. The survey asked respondents to rate (a) their satisfaction with the learning module components, including the content and (b) the perceived impact the learning has had on their practices and patients. The psychometric properties (Chronbach’s alphas) of the satisfaction and impact scales ranged from .82 to .94.

**Results:**

Evaluation findings from the first three years of the PSP indicated consistently high satisfaction ratings and perceived impact on GP practices and patients, regardless of physician characteristics (gender, age group) or work-related variables (e.g., time worked in family practice). The Advanced Access Learning Module, which offers tools to improve office efficiencies, decreased wait times for urgent, regular and third next available appointments by an average of 1.2, 3.3, and by 3.4 days across all physicians. For the Chronic Disease Management module, over 87% of all GP respondents developed a CDM patient registry and reported being able to take better care of their patients. After attending the Adult Mental Health module: 94.1% of GPs agreed that they felt more comfortable helping patients who required mental health care; over 82% agreed that their skills and their confidence in diagnosing and treating mental health conditions had improved; and 41.0% agreed that their frequency of prescribing medications, if appropriate, had decreased. Additionally for the Adult Mental Health module, a 3–6 month follow-up survey of the GPs indicated that the implemented changes were sustained over time.

**Conclusion:**

GP and medical office assistant participant ratings show that the PSP learning modules were consistently successful in providing GPs and their staff with new learning that was relevant and could be implemented and used in “real-GP-time”.

## Background

Continuing medical education is an international concern [1]. It takes on many configurations and approaches including: using written materials with follow-ups [2], videoconferencing [3], distance learning [4], peer review groups [5], in practice learning [6], and web based learning [7]. However, the most common approaches focus on interactive teaching workshops [8-12]. In most of these cases, the educational interventions are short and have small numbers of participants. There are also examples of longer (3 yr) educational initiatives [13,14], and train-the-trainer initiatives [15,16]. Many European countries, and particularly the Dutch, have developed quality circles and peer review, peer-to-peer, groups for quality improvement. However, these initiatives are usually sponsored by physicians themselves and/or their respective organizations. They do not appear to be part of an active collaborative initiative between government and the medical profession [17]. Aside from the Australian General Practice and Education Training Program, with regional practice training providers [18], there are few, large-scale (provincial/state/national level) continuing medical education initiatives, or initiatives that are based on government and physician collaboration. Thus, it appears that the approach taken to continuing medical education in British Columbia, Canada, in the form of the Practice Support Program (PSP), is relatively unique.

The PSP offers peer-to-peer training to physicians and their office staff on a variety of topics, ranging from office management to specific clinical topic areas. The training modules are supported by dedicated regional staff and are conducted by general practitioner (GP) and medical office assistant (MOA) champions. (Medical office assistants typically have a one-year college based training program, but they may also be trained on the job. They should be considered to be support staff with medical training. They may take vital signs, provide patient self-management support and so on.) Remuneration is provided to participating GPs and MOAs. GPs also receive continuing education credits. This paper briefly describes the first five learning modules offered by the PSP and reports on the findings from evaluation surveys of GP and MOA participants who completed the learning modules.

### The practice support program (PSP)

The PSP is a novel program that was developed in response to feedback received from some 1,000 GPs across British Columbia during a consultation process conducted in 2004–05. The consultation found that GPs asked to be valued, to be paid fairly, to be trained and to be supported. The establishment of the PSP responded to three of these four priority areas. The Full Service Family Practice Incentive Program (FSFPIP) which provides additional incentive payments, responded to the issue of fair payment. The consultation was undertaken by the General Practice Services Committee (GPSC), which was created in 2002 to “reform” family medicine in BC [19,20]. Membership on the GPSC consists of representatives from the Ministry of Health, the British Columbia Medical Association (BCMA), the Society of General Practitioners of British Columbia, and regional health authorities. Funding is provided by government in accordance with a formal Ministry and BCMA joint Agreement.

The PSP is a practice change program that consists of a number of continuing education learning modules for GPs and their MOAs (see http://www.gpscbc.ca/psp/learning). The modules were, and new ones continue to be, designed by content experts with input from GPs to make the content useful and “doable in real GP time”. The content areas of the modules include clinical topics as well as practical office management re-tooling. The first five learning modules were: advanced access (AA); chronic disease management (CDM); patient self-management (PSM); group medical visits (GMV); and adult mental health (with a focus on depression) (AMH). The first four modules were implemented in the fall of 2007, and have undergone some revisions in delivery (viz., for a short period the content of the CDM module became combined with the content of the PSM and/or GMV modules). The success of the first four modules during the first year of the PSP has been documented in an earlier publication [21]. The AMH learning module was added to the roster of modules in the summer of 2009 and also met with early success [22]. In addition, a train-the-trainer process was introduced with the AMH module to train the GP and MOA peer trainers (the “champions”). Training of peer champions takes place during Train-the-Trainer (TTT) phase for each new topic area (module), and consists of one or two Train-the-Trainer sessions to familiarize the peer champions with the content and the clinical tools. The first session (TTT1) is followed by an action period when the peer champions try the materials and the tools in their own office practice to ensure that they understand how to use them and feel comfortable with them. The peer champions return to a second TTT session to report on their experience with the content/tools, recommend any changes to the content, and to receive extra training in adult education principles, facilitation skills and presentation skills to ensure they feel confident leading the learning sessions in their own communities. Having peer physician champions co-deliver learning sessions contributes to the credibility of the content and tools presented by “a GP just like me” as the champions are able to describe how much time and effort will be required by the practice.

Regional Support Team (RST) members, who assist GPs and MOAs at the local level, attend the same sessions. They also receive additional training during provincial learning sessions that focus on quality improvement methods, coaching skills and the human dimension of change. Monthly support calls for the training teams are also organized by the PSP central office to provide a provincial forum for sharing, learning and asking clinical questions.

The format of all learning modules is consistent across topic areas and is team-based, dynamic and interactive. Each learning module consists of pre-work, three half-day learning sessions interspersed with action periods of about two months where participants try out the new learnings in their practices with the intent of embedding the changes into everyday routine practice. The main content (which varies across modules) is usually taught in the first learning session. The content is practiced by GPs in the first action period. Additional material is taught at the second session and all learnings are practiced during the second action period. The third learning session serves as an overall review of learnings and experiences. GPs share their experiences with their colleagues and find this type of interaction to be particularly helpful and positive. Throughout the learning module, participants are supported by the PSP regional support team members. Each teaching team consists of: the peer GP and MOA “champions” (the instructors of the learning modules who have had special training on the content themselves); RST staff (who organize, coordinate and facilitate the learning sessions and provide support during the action periods); and, if the content area is clinical, team member(s) from the relevant specialty and/or allied health care profession (to serve as content experts/consultants).

The PSP delivery method is based on the Institute for Health Improvement (IHI) Breakthrough structured learning series approach but has been significantly adapted to suit the local BC environment. For example, the learning sessions are much shorter (half-day) and there are two action periods, as we found that physicians would not attend longer and more numerous learning sessions. The IHI Model for Improvement forms the theoretical basis for making practice changes, as we have found that small, rapid cycle tests of change work well in physician practices. Physicians can see improvements very quickly, but if the change is unsuccessful, the practice will not have invested too much time and effort.

Participation in the learning modules is voluntary and GPs are encouraged to invite their MOAs to attend and actively participate in implementing the new ideas. The overall engagement level in the PSP after 5 years is currently at 67% of all actively practicing family physicians in the province. Physicians are reimbursed for time taken out of their practice not seeing patients, and the total payment amount for a topic module per GP is $2,900. Finally, the Practice Support Program is accredited at the highest level for family physicians as well as for specialist physicians.

New modules are currently in various stages of development, but are not reported on here. In this paper, we present the evaluation findings for the five modules based on the first three years of the PSP.

### Evaluation of the practice support program (PSP)

Evaluation of the PSP learning modules has been an integral feature of the PSP since its inception and has been evolving and expanding over time. For the first four PSP learning modules, we conducted outcome evaluations to assess whether the program was meeting its objectives [23]. Surveys were administered to GP and MOA participants at the completion of the learning module. The surveys asked for GPs' ratings of: (1) satisfaction with a number of aspects of the learning modules and (2) perceived impact on their work environment and their patients. For the Adult Mental Health learning module, a more extensive outcome evaluation that included a three-to-six month follow-up survey of the GP participants was added to the end of module survey. The follow-up survey assessed the longer-term impacts and sustainability of the newly learned skills.

Each module was evaluated separately and on an annual basis, providing continuous feedback to the module developers. In this report, we present the findings aggregated across the program’s first three years of operation. We found that the findings were very similar across the three years, which allows us to pool the results, but also provides “replications” of the findings across time as well as across the different modules. These natural replications further validate the effectiveness of the program in re-engaging family physicians across the province.

## Method

### Participants

GPs and MOAs who completed each learning module, from inception to March 31, 2011, were asked to complete an anonymous end of module survey. The numbers of respondents, by module topic, are shown in Table 1. For the four initial learning modules, response rates for the GPs were 26.0% in 2008–09, 48.8% in 2009–10, and 36.2% in 2010–2011. The response rates for the MOAs, over the three year period, were 21.3%, 49.8% and 24.4%. For the Adult Mental Health module, the GP response rates were 60.2% in 2009–10 and 57.0% in 2010–11 and the MOA response rates were 39.7% in 2009–10 and 37.5% in 2010–11. (The response rates are our best estimates based on the number of questionnaires the research team provided to the RSTs for administration at the last learning session. The estimated response rates reported here are thus likely to be underestimates as enough surveys were sent out for all potential attendees.) The GPs’ response rate on the 3–6 month follow-up survey for AMH was 19.6%.

**Table 1:** GP and MOA attendance at the five different PSP learning modules

**Learning Module Topic Area**	**2008-09**^**1**^		**2009-10**		**2010-11**		**TOTAL (Across Years)**	
	**N**	**%**^**2**^	**N**	**%**	**N**	**%**	**N**	**%**
**GP Respondents**								
Advanced Access	107	49.8	45	15.4	5	1.3	157	17.7
Chronic Disease Management	55	25.6	92	31.5	32	8.4	179	20.2
Patient Self-Management	45	20.9	37	12.7	12	3.2	94	10.6
Group Medical Visits	25	11.6	48	16.4	26	6.8	99	11.2
Adult Mental Health^3^	n/a	n/a	136	46.6	329	86.6	465	52.4
AMH 3–6 Month Follow-up	n/a	n/a	37	n/a	74	n/a	111	n/a
**Yearly Total Across Topics**^**4**^	**215**	**-**	**292**	**-**	**380**	**-**	**887**	**-**
**MOA Respondents**								
Advanced Access	81	50.3	25	17.7	3	2.9	109	26.9
CDM	42	26.1	66	46.8	18	17.5	126	31.1
Patient Self-Management	31	19.3	21	14.9	6	5.8	58	14.3
Group Medical Visits	20	12.4	30	21.3	14	13.6	64	15.8
Adult Mental Health	n/a	n/a	37	26.2	74	71.8	111	27.4
**Yearly Total Across Topics (4)**	**161**	**-**	**141**	**-**	**103**	**-**	**405**	**-**

Notes:

^1^ The years are April 1 to March 31.

^2^ The % is of the Yearly Total; entries in this column give the distribution by topic area and sum to more than 100% (see Note 4 below).

^3^ This module was implemented in the Fall of 2009; the 3–6 month follow-up surveys began in Nov 2009.

^4^ The total number of respondents across topic areas is the number of GPs/MOAs in the given year and does not equal the sum of the number of respondents across topic areas. This is because some participants attended learning modules that presented combined topic areas and they are counted in each topic area.

Of the 887 GPs who completed the end of module surveys: 55.3% were men, 36.8% were 40–49 years old with an additional 33.4% being 50–59 years old. Most (74.9%) worked full time. Large percentages worked in group practices, with 34.1% in small group (3–4 physician) and 30.6% in large (5 or more physician) practices, whereas 18.0% worked solo and 16.8% worked with one other GP. Some 75.3% of physicians had attended more than one of the PSP modules.

All but 4 of the 405 MOAs (99.2%) were women, with a relatively even distribution across age groups: 21.3% were 29 or younger; 24.2% were 30–39 years old; 27.5% were 40–49 years old; and 26.7% were 50 years or older. Over half (50.4%) had worked in a family practice for 10 years or less and 73.0% were full-time. Slightly larger percentage (29.3%) worked in solo practices, compared with 18.7% in two-physician practices, and 26.2% and 25.6% in small group and large group practices respectively.

For the Adult Mental Health module, 111 GPs completed the 3–6 month follow-up survey. This sub-group was similar to the overall group of GPs: 50.5% were men, 57.8% were 50 years or older, 77.1% worked full-time, 17.6% worked solo and 21.3% worked with one other physician while 61.1% worked in group practices.

Initially GPs who dropped out were also surveyed but it was found that the reasons for dropping out were not related to the quality of the learning modules *per se*. Rather, they had to do with GPs’ time constraints, moving to another location, already being familiar with the material and so on. Furthermore, while GPs may have dropped out they often came back to take the module at a later date. The overall drop out rate was seen as modest. Thus, there did not appear to be a need to continue these surveys. However, if circumstances change they can always be re-implemented at a later date, as appropriate. Finally, as this project was an evaluation of a quality improvement initiative it did not require an ethics review.

### Procedure

A paper version of the end of module survey was administered at the end of the last learning session by RST members. GPs and MOAs had the option of returning the completed survey to the team member in a sealed envelope for return to the evaluation team in bulk, or they could mail the completed form directly in the addressed postage-paid envelope provided.

The 3–6 month follow-up survey was mailed to all physicians who had completed the Adult Mental Health learning module 3 to 6 months following their completion of the learning module. To ensure anonymity, the mailing was done by the BCMA, and the GPs were asked to return the completed questionnaire directly to the evaluation team.

### Survey questionnaires

The end of module surveys for the GP participants consisted of four sections of questions, three of which were common to all modules. Section 1 (8 items) asked for socio-demographic information. Section 2 (22 items), the Satisfaction Scale, asked for the participants’ ratings of satisfaction with five different aspects of the learning module – overall impressions, the learning sessions, the action periods, goals and measures, and the general PSP program. Section 4 (16 items) asked participants to rate the perceived impact of the new learnings on their practices. Section 3 asked questions specific to the content of each learning module. The number of items ranged from 8 to 15. Respondents were also given an opportunity to provide written comments.

The psychometric properties of the scales have been well established over the course of the evaluation. Because the Adult Mental Health (AMH) module had a different time course, we report its statistics separately. The psychometric properties were similar across the modules and across time, creating natural replications of the psychometric assessments of these scales and providing evidence of their stability across content areas and time. Here we report the psychometric properties pooled across the first three years of the survey.

The internal consistencies of the two scales and five subscale scores were assessed using Cronbach’s alpha. The Cronbach alphas for the 22-item Satisfaction Scale for the original four modules and the Adult Mental Health module were: .89 and .86 respectively (subscale scores ranged from .60 to .88) for the GPs; and .82 and .84 (ranging from .54 to .82 on the subscales) for the MOAs. The corresponding Cronbach’s alphas for the 16-item Perceived Impact scale were .94 and .90 for the GPs and .84 and .84 for the MOAs. The Cronbach alphas for the content scales were: Chronic Disease Management (CDM) = .89; Patient Self Management (PSM) = .85; Group Medical Visit (GMV) = .83; and AMH = .87 for the GPs (the MOAs did not have different content subscales). Items for the Advanced Access Module did not form a scale, but asked for various numerical estimates (see below).

### Analyses

The Satisfaction Scale, Perceived Impact Scale and Content Scale scores were computed for each survey participant. In the first set of analyses, experiences with the learning modules were examined as a function of the type of learning module and the type of participant (i.e., the participants’ socio-demographic variables). For each scale we conducted: a one-way analysis of variance (ANOVA) with type of learning module as the independent variable and the scale score as the dependent variable; and two-way factorial ANOVAs with the type of learning module and each of the seven socio-demographic variables as the two independent variables. Because eight statistical ANOVAs were conducted for each scale, we used the Bonferroni correction to protect against Type I error and set our significance level for each ANOVA at .006 (=.05/8). Any statistically significant main or interaction effects were explored with post-hoc tests, using the Bonferroni correction for Type I error for each set/family of post-hoc comparisons. For a finer-grained look at the participants’ experiences with the learning modules, we examined the distributions of responses on the individual scale items (descriptively only).

## Results and discussion

We report the results in four sections: attendance at the learning modules, satisfaction ratings, overall practice specific impacts, and module-specific impacts.

### Attendance at the learning modules by topic area

The percentages shown in Table 1 indicate that the relative interest in the various topic areas changed across the first three years of the PSP. For both GPs and MOAs, the Advanced Access module was the most highly attended module in the first year, with 49.8% of all GPs and 50.3% of all MOAs choosing to attend this module. When the Adult Mental Health module became available in the fall of 2009, it saw a strong uptake by the GPs, both in terms of actual numbers of attendees as well as the relative percentage. As a consequence, the chronic disease management, and especially the patient self management and group medical visits topic areas, saw a general decrease in attendance. (As noted in the introduction, CDM was combined with the PSM and/or GMV topic areas for a period of time as the PSP evolved. To streamline our findings, we included the responses of participants who completed these combination learning modules in each of the constituent topic areas).

### Satisfaction with the learning modules

For the GP respondents, a one-way ANOVA on the total Satisfaction Scale scores indicated a statistically significant effect of type of module, F(4, 913) = 27.907, p<.001, MSE = 84.2, η^2^ = .109. Post-hoc tests indicated that the average satisfaction rating for the AMH module was statistically higher (M = 93.2, SEM = 0.44) than the average satisfaction ratings for the other four modules which did not differ statistically from each other (means ranged from 86.2 to 87.5, and SEMs ranged from 0.65 to 0.96).

In assessing whether any of the socio-demographic or practice based variables were related to the GPs’ satisfaction ratings, we found statistically significant differences in satisfaction only by geographic region in the province, F(4, 894) = 10.358, p<.001, MSE = 81.3. This result was found across all five module topic areas, and did not interact with the topic (F (15, 894) = 1.232, p = .241). None of the GP characteristics (gender, age group) nor work-related variables (time worked in family practice, type of practice [solo, two-physician, small group or large group practices], full- versus part-time work schedule) were related to the satisfaction ratings (all p-values > .012 and greater than our Bonferroni-corrected cut-off of .006).

**Figure 1:**
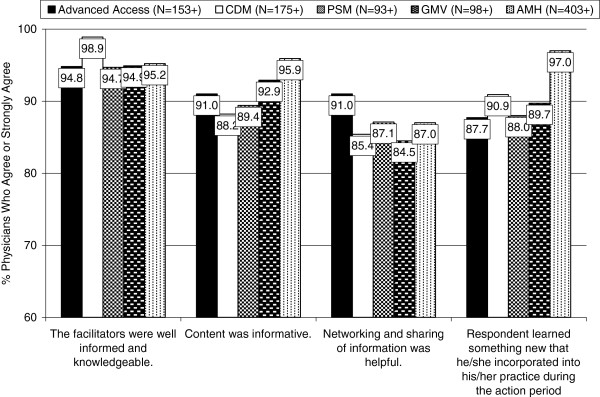
GPs’ Ratings of Overall Satisfaction With the Five PSP Learning Modules.

Ratings on the individual Satisfaction Scale items were consistently high, although slight variations across the different learning modules were observed. Examples of four key items (of 22 items) are shown in Figure 1. Of particular note is the almost unanimous agreement by GP respondents that the facilitators of all learning modules (but especially CDM) were well informed and knowledgeable (over 94% agreement across all modules). Satisfaction varied somewhat across module topics areas, although all modules were rated highly. For example, whether the GPs learned something new that they incorporated into their practice during the action period was lower for those attending Advanced Access (87.7%) compared with those attending the Adult Mental Health (97.0%). Generally, however, responses on most items were similar and consistently high, indicating satisfaction with all modules.

**Figure 2:**
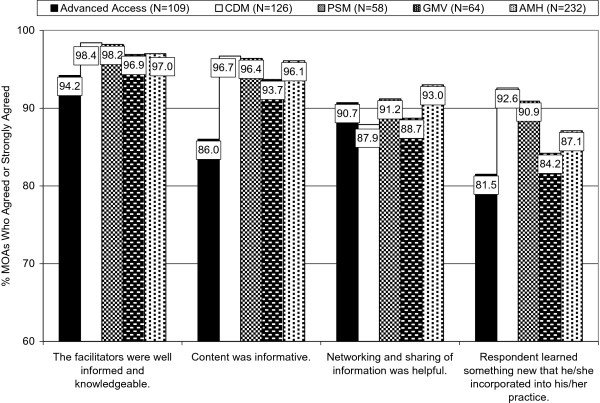
MOAs’ Ratings of Overall Satisfaction With the Five PSP Learning Modules.

The satisfaction ratings of the MOA respondents generally paralleled those of the GPs. There were no statistically significant group differences in the MOAs’ ratings by type of learning module or any of the socio-demographic variables. As was the case with the GP respondents, large percentages of the MOA respondents rated their satisfaction with the learning modules highly. Figure 2 shows the MOAs’ ratings on the same sample of items as were shown for the GPs. The percentages of MOAs who agreed or strongly agreed with these four key items (of 16 items) on the Satisfaction Scale are even higher than those of the GPs. The one notable exception is the item asking whether the respondents learned something new that they incorporated into their own practice: Although still high at 87.1%, the percentage of MOAs in the Adult Mental Health learning module was about 10% lower than for the GPs.

### Perceived impact on the GPs’ practices

A one-way ANOVA on the GPs’ Perceived Impact scale scores also indicated a statistically significant effect of type of module, F(4, 822) = 61.372, p<.001, MSE = 89.1. Post-hoc tests found that average perceived impact of the Adult Mental Health module was statistically higher (M = 68.4, SEM = 0.49) than that of the other modules.

As we observed with the Satisfaction scale scores for the GPs, Perceived Impact scores varied by geographic region, F(4, 822) = 61.37, p<.001, MSE = 89.12, η^2^ = .230. In addition, perceived impact also varied slightly but statistically significantly by the GPs’ gender (F(1,813) = 9.26, p=.002, MSE = 88.84), with male GPs rating the perceived impact higher (M = 63.3) than female GPs (M = 61.3). The perceived impact ratings did not differ by GPs’ age group, time in family practice, nature of the practice, nor full- versus part-time work schedules (all p-values >.056).

**Figure 3:**
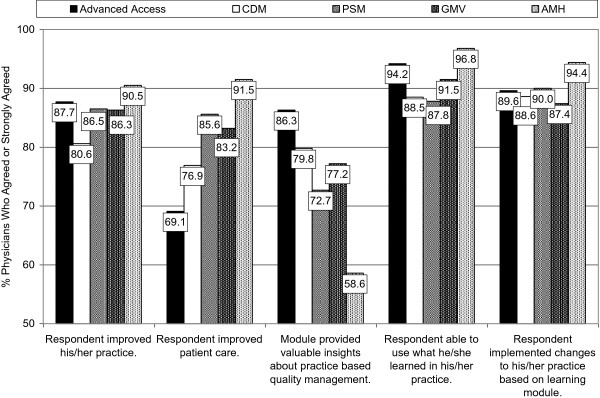
GPs’ Ratings of Perceived Impact of the Five PSP Learning Modules.

Five individual items of particular interest (of 19 items on the Perceived Impact Scale) are shown in Figure 3. Over 80% of GPs agreed or strongly agreed that attending the module improved their practice. Different levels of agreement of the GPs were observed across the five different module topics. For example, 91.5% of GPs who attended the Adult Mental Health module agreed or strongly agreed that attending the module had improved patient care, compared with 69.1% of GPs who attended the Advanced Access module. In contrast, 86.3% of GPs who attended Advanced Access agreed or strongly agreed that attending had provided them with insights about practice based quality management, compared with 58.6% of GPs who attended Adult Mental Health.

**Figure 4:**
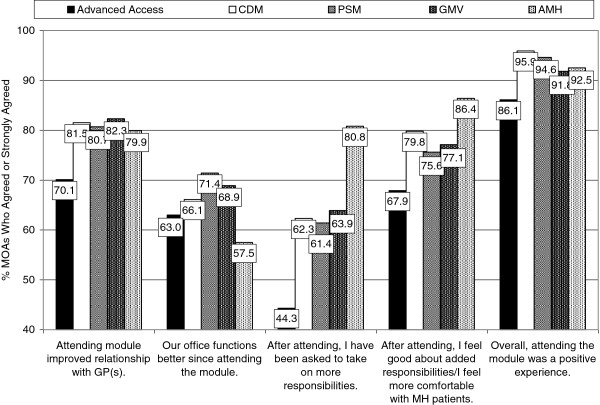
MOAs’ Ratings of Perceived Impact of the Five PSP Learning Modules.

The perceived impact ratings by the MOAs are shown in Figure 4 for a sample of five items (of 8 items) on the Perceived Impact Scale. These items were different from those on the GP survey. But as was observed with the GPs, the different learning modules had differential impacts on different aspects of the MOAs’ work environments. For example, fewer MOAs (44.3%) were asked to take on more responsibilities following their attendance of the Advanced Access module, one of the earliest modules offered, compared with 80.8% of those who attended the Adult Mental Health, with parallel ratings about how they felt about taking on these additional responsibilities. In fact, some 40.3% to 80.8% of MOAs noted that they had been asked to take on more responsibilities, and 67.9% to 86.4% said that they felt good after taking on additional responsibilities (see Figure 4). For large percentages of MOAs (86% to 96% across the five modules) attending the learning module was perceived as an overall positive experience. Furthermore, for most MOAs (over 70% overall), attending the module with their GP had improved their relationship with their GP.

### Module-specific impacts on GP practices

The evaluation has evolved along with the evolution of the learning modules over time. For the first four modules an end of module survey was used (this practice has continued over time). For the Adult Mental Health Module, a supplement was added to the end of module survey to obtain additional information. The supplement survey was also sent out 3–6 months after the completion of the learning module to access the sustainability of the learnings, and the impact on GP practices over time. For the newly implemented end of life module, a baseline survey was added which is to be administered at the beginning of the first learning session. For future learning modules we shall also be adding a patient experience survey to obtain the patients’ perspectives’.

It was not possible to compare the participant characteristics of module attendees to all GPs or to compare the characteristics of module attendees who did, and did not, complete the surveys, as comparable data were not collected. However, it is quite clear that the satisfaction with, and the impact of, the learning modules were, on average, quite positive across all GPs who attended all modules.

#### Advanced access

This module offered office management tools to improve office efficiencies. Table 2 shows the changes in wait times (in days) on urgent, regular and third next available appointments, based on estimates given by all GP respondents. The estimates show that, on average, wait times for all respondents decreased by 1.2 days for urgent appointments (t(142) = 5.18, p<.001), 3.3 days for regular appointments (t(140) = 8.09, p<.001), and by 3.4 days for third next available appointments (t(127) = 36.68, p<.001). Not all GPs, however, reported a decrease in their wait times following their attendance at the learning module. Specifically, for urgent appointments, only 51.0% of GPs indicated a decrease, whereas for regular and third next available appointments, 70.2% and 75.8% of GPs indicated a decrease. For these subsets of GPs, the average decreases were 2.4 days, 4.9 days and 4.6 days for urgent, regular and third next available appointments, respectively (and all p-values < .001). In addition, of all GP respondents: 67.3% reported having reduced their backlog; 66.9% were able to start and end their work days on time; 24.2% were able to take more time off; and 37.1% had increased their panel size as a result of attending this module.

**Table 2:** Impact of attending the advanced access learning module on three types of patient appointments

**Type of appointment**	**Wait times, in days, for Appointment:**		**Difference (Mean Number of Days)**
	**Mean (Standard Error of the Mean)**		
	**Before the module**	**After the module**	
**All GP Respondents (N=143)**			
Urgent	1.64 (0.23)	0.43 (0.05)	1.21*
Regular	6.09 (0.45)	2.75 (0.26)	3.34*
Third Next Available	5.20 (0.55)	1.80 (0.24)	3.40*
**GP Respondents Who Decreased Wait Times (N=73)**			
Urgent	2.72 (0.74)	0.33 (0.10)	2.39*
Regular	7.12 (1.14)	2.21 (0.43)	4.91*
Third Next Available	6.10 (1.37)	1.51 (0.34)	4.59*
* p<.001			

#### Chronic disease management (CDM)

A large percentage of GP respondents agreed or strongly agreed that having attended this module: had allowed them to take better care of their patients (87.6%); had helped them identify patients with chronic disease (84.2%); prompted the development of a CDM patient registry for their practice (88.6%); and prompted them to actively consider CDM guidelines in care delivery (84.7%). Slightly lower percentages, but still the majority of GPs, agreed that attending the module made patients seem satisfied with, and/or engaged in, their care (65.0%) and increased the GP’s satisfaction with work (61.3%).

#### Patient self-management (PSM)

Large percentages of GPs agreed or strongly agreed that: they felt comfortable with helping their patients adopt self-managed care (87.6%); they would make PSM an ongoing part of their practice (86.5%); and they were partners with their patients in their patients’ care (92.0%). Moderate levels of agreement were obtained from GPs about their patients seeming to like self management (69.3%), being more involved in self-management (69.7%) and being satisfied with their care (61.8%). Also, 65.2% of GPs agreed or strongly agreed that attending this module had increased their work satisfaction; however, a few GPs (23.6%) noted that offering self-managed care was too time consuming.

#### Group medical visits (GMV)

Some 73.9% of GPs were comfortable conducting group visits after completing this module. Most GPs agreed that their patients liked the peer learning (78.8%), liked the patient self-management support provided by the group visits (79.8%), and were satisfied with their care (76.2%). Some 77.4% of the GPs agreed that group visits allowed them to use a team-based approach to care, although 45.9% agreed that scheduling them was difficult. Some 60.0% indicated that they planned to make group visits an ongoing part of their practice.

#### Adult mental health (AMH)

On completing the AMH module, 94.1% of GPs agreed that they felt more comfortable helping patients who required mental health care. Attending the module had enhanced their skills (86.6% of GPs agreed) and their confidence (82.1%) in diagnosing mental health conditions, and their skills (88.8%) and confidence (87.0%) in treating mental health conditions. Attendance increased work satisfaction for 68.2% of GP respondents.

GPs agreed or strongly agreed that attending the module enhanced their skills in: conducting a diagnostic assessment interview (86.8%); offering and coaching the Antidepressant Skills Workbook [24] (81.4%); offering and encouraging the use of the Bounce Back Program [25] (91.4%); and coaching patients on a variety of cognitive behavioural and interpersonal skills (82.7%). Attending the module had improved their ability to develop appropriate care plans (79.2% of GPs agreed), increased care partnerships of GPs with their patients (91.8%), and increased patients’ engagement in self-management of mental health concerns (60.9%). Furthermore, 41.0% of GPs indicated that they had decreased their frequency of prescribing medications, if appropriate, while only 20.8% indicated they had increased their prescribing.

Furthermore, data from the Supplement Survey indicate that 60.2% of the GPs reported high or very high success in implementing the newly learned tools and skills into their practices, and 73.2% reported they will continue to use them. Some 22.9% of the GPs reported a high degree of MOA involvement, with an additional 40.0% indicating a moderate involvement. The impact of the AMH module on their patients was rated as positive or very positive by 94.5% of GP respondents, with the remaining GPs indicating “no impact”. Specifically with regard to helping their patients remain at work or return to work following cognitive behavioural interventions (as compared to without): 89.2% of GPs reported their patients were better or much better able to continue to work; and 78.4% of GPs reported their patients were better or much better able to return to work (the remaining GPs reported “no change”).

Findings from the 3–6 month follow-up supplement survey indicated that the new learning was sustained. Comparisons of self-ratings of confidence, at the completion of the module and at follow-up, are shown in Table 3. Confidence remained at similar levels or increased slightly over time on many dimensions. Of particular note is item (m) that asks for their overall assessment of their confidence in providing quality mental health care to their patients: At the end of their training, the average rating was 1.71 (on a four-point scale where 1 was very confident, 2 was somewhat confident, 3 was not very confident, and 4 was not at all confident); this dropped (indicating an increase in the confidence rating) to an average confidence rating of 1.46. In addition, almost all responding GPs (99.1%) indicated that they were confident in their ability to provide quality mental health care to their patients. (A caveat to this finding is that this result may be, at least in part, due to a selection bias, as it is possible that primarily those GPs who were successful in using the tools and skills responded on the 3–6 month follow-up survey.)

**Table 3:** Long-term maintenance of GPs’ confidence in providing mental health care

	**Confidence ratings at end of module (N≥294)**		**Confidence ratings 3–6 Months after completion (N≥106)**	
	**Mean**^**1**^	**%**^**2**^	**Mean**^**1**^	**%**^**2**^
**Respondent’s Confidence in his or her ability to**:				
(a) diagnose depression	1.23	99.7	1.19	100
(b) screen for addictions	1.74	92.4	1.67	94.5
(c) screen for other mental health conditions	1.83	91.8	1.73	96.3
(d) treat depression	1.43	99.0	1.28	100
(e) treat other mental health disorders	1.99	85.2	1.93	87.2
(f) prescribe medications for mental health conditions	1.67	95.0	1.61	95.5
(g) assess patients’ problems and strengths	1.79	91.0	1.66	92.7
(h) develop systematized care plans for patients where a mental health care plan is not appropriate	2.07	77.5	1.86	84.0
(i) create a mental health care plan fitting Medical Service Plan [provincial program] guidelines	1.87	83.4	1.69	89.8
(j) engage mental health patients in a range of interventions (e.g., cognitive behavioural and interpersonal skills training)	1.97	81.4	1.90	82.6
(k) offer and coach the Antidepressant Skills Workbook	1.95	80.2	1.83	85.0
(l) offer and support the Bounce Back program	1.54	92.1	1.56	91.7
(m) In general, how confident are you in the quality of mental health care you provide to your patients?	1.71	96.3	1.46	99.1
**Respondent’s confidence in her or his knowledge/awareness of:**				
(a) non-pharmacological interventions (cognitive behavioral skills such as activation, relaxation, negative thinking)	1.90	86.8	1.76	91.7
(b) regional mental health resources for mental health patients	2.05	82.8	1.87	88.1

**Notes**:

^1^ Lower means indicate higher confidence level rating. This was a 4-point rating scale with 1 indicating “very confident”, 2 indicating “somewhat confident”, 3 indicating “not very confident” and 4 indicating “not at all confident”.

^2^ Percentage of GPs who responded “very confident” or “somewhat confident”.

## Conclusions

The evaluation of the first three years of the PSP showed that both GP and MOA participants rated their satisfaction with most aspects of the learning modules consistently highly. Both GPs and MOAs also rated the perceived impact of the new learnings positively overall, but as would be expected, some topic areas resulted in differential impacts on their practice/work environment and their patients. For example, operational changes to the Chronic Disease Management module in which other topic areas were added into the Chronic Disease Management module, lengthened the learning module significantly. This was discouraging to some GPs. Attending the Advanced Access learning module provided valuable insights about practice-based quality management and improved office efficiencies, but did not particularly improve patient care. Conversely, the Adult Mental Health learning module was viewed as substantially improving patient care while not impacting the practice based quality management aspect. In general, the Adult Mental Health Module was seen to be of particular relevance and interest to GPs, at least some of whom did not feel that they were adequately trained to help mental health patients in their office. Very high percentages of GPs reported using what they learned in the modules in their own practices, ranging from 87.8% of GPs using learning from the Patient Self-Management module to 96.8% using new skills and tools from the Adult Mental Health module. These high rates of uptake of the new tools and skills indicate that the content was in fact highly relevant to the GPs and “doable in real GP time.”

The partnership between the government and the medical association is seen as critical to the success of the program. The PSP complements the financial incentive payments with practice support and clinical tools for difficult patient populations such as patients with mental health disorders, and those requiring end of life care. Housing the program within the medical association lends credibility to the program as physicians trust their professional association. The BCMA speaks the same language as GPs, and is sensitive to the realities of physician practices and patient relationships. It also puts the concept of “change and quality through collaboration” at the intersection of front line physicians, their association, and their funder (the Ministry of Health).

The positive results reported in this paper, based on the feedback received from the beneficiaries of the program, may be of interest to other jurisdictions where change management and quality improvement initiatives are being considered. As well as offering topics of high interest to physicians, the PSP brings together the key elements of peer-to-peer training, active involvement of the physicians’ office staff, dedicated program support staff who ensure smooth delivery and accountability by the participants, and opportunities for participants to meet, interact, network and share experiences. It is these elements that together create the positive change that the PSP has had on physicians in British Columbia.

### Next steps

Looking forward, the PSP continues to work on improving existing modules and introducing new modules, on a range of topics including end of life care and child and youth mental health. In the intermediate term the plan is to reach 75% of the actively practicing family physicians during the current fiscal year (ending March 31, 2013). The program is also in the process of delivering an in-practice coaching development program for the PSP regional support team members to allow them to go ‘deeper’ into each practice and support the longer term sustainability of practice improvements. The program also intends to involve specialist physicians in the next series of new modules. Specific initiatives to do so are currently under consideration.

## Competing interests

Dan MacCarthy and Liza Kallstrom were involved in developing the Practice Support Program and are salaried staff of the British Columbia Medical Association (BCMA). The PSP is one of the General Practice Services Committee’s (GPSC) initiatives. Helena Kadlec and Marcus Hollander are with Hollander Analytical Services Ltd. To ensure the independence and objectivity of evaluations conducted by Hollander Analytical Services Ltd, which are funded by the GPSC, the BC Ministry of Health and the BCMA have signed an agreement, on behalf of the GPSC, that guarantees the integrity, objectivity and independence of any evaluations conducted for the GPSC by Hollander Analytical Services Ltd. Thus, there are no competing interests.

## Authors’ contributions

DMC and LK developed and implemented the Practice Support Program (PSP). They ensured that the evaluation was consistent with the design and operation of the PSP. They also reviewed and provided helpful corrections of fact and comments on the draft text of the paper. MH designed and implemented the PSP evaluation and reviewed all analyses conducted and final reports. HK assisted with instrument design for the Adult Mental Health Surveys, conducted the analyses presented in this paper and prepared draft versions of the manuscript for review. All authors read and approved the manuscript.

## Authors’ information

DM is the Executive Director, Practice Support and Quality, British Columbia Medical Association (BCMA). This is a senior position which includes the Practice Support Program (PSP). DM was, and is, the senior official responsible for overseeing the development of the PSP and its learning modules.

LK reports to DM at the BCMA and has been the driving force in designing and implementing the PSP learning modules across the province, and establishing and working with the Regional Support Teams which assist and facilitate the conduct of the learning modules.

HK is the Senior Scientist with Hollander Analytical Services Ltd. She holds a PHD in quantitative psychology, and teaches at the University of Victoria on a sessional basis.

MH is president of Hollander Analytical Services Ltd, a national health services and policy research company, headquartered in Victoria, BC. MH holds a PHD in human and social development, and leads large-scale research projects and program evaluations in health policy. He is a member of The EvidenceNetwork.ca, a national consortium of experts on knowledge transfer in health policy related research and development.

## Pre-publication history

The pre-publication history for this paper can be accessed here:

http://www.biomedcentral.com/1472-6920/12/110/prepub
